# Crystal structure of *trans*-di­aqua­bis­(4-cyano­benzoato-κ*O*)bis­(*N*,*N*-di­ethyl­nicotinamide-κ*N*)zinc(II)

**DOI:** 10.1107/S2056989016013815

**Published:** 2016-09-05

**Authors:** Nurcan Akduran, Hacali Necefoğlu, Ömer Aydoğdu, Tuncer Hökelek

**Affiliations:** a SANAEM, Saray Mahallesi, Atom Caddesi, No:27, 06980 Saray-Kazan, Ankara, Turkey; b Department of Chemistry, Kafkas University, 36100 Kars, Turkey; c International Scientific Research Centre, Baku State University, 1148 Baku, Azerbaijan; d Department of Physics, Hacettepe University, 06800 Beytepe, Ankara, Turkey

**Keywords:** crystal structure, zinc complex, benzoate, nicotinamide derivatives

## Abstract

In the title complex, [Zn(C_8_H_4_NO_2_)_2_(C_10_H_14_N_2_O)_2_(H_2_O)_2_], the Zn^II^ cation, located on an inversion centre, is coordinated by two water mol­ecules, two 4-cyano­benzoate (CB) anions and two di­ethyl­nicotinamide (DENA) ligands in a distorted N_2_O_4_ octa­hedral geometry. In the mol­ecule, the dihedral angle between the planar carboxyl­ate group and the adjacent benzene ring is 9.50 (14)°, while the benzene and pyridine rings are oriented at a dihedral angle of 56.99 (5)°. The water mol­ecules exhibit both an intra­molecular hydrogen bond [to the non-coordinating carboxyl­ate O atom, enclosing an *S*(6) hydrogen-bonding motif, where O⋯O = 2.6419 (19) Å] and an inter­molecular hydrogen bond [to the amide carbonyl O atom, enclosing an *R*_2_^2^(16) ring motif, where O⋯O = 2.827 (2) Å]; the latter lead to the formation of supra­molecular chains propagating along the [110] direction.

## Abstract 2

In the crystal, the title centrosymmetric Zn^II^ complex mol­ecules are linked by O—H⋯O hydrogen bonds into supra­molecular chains propagating along the [110] direction.

## Chemical context   

Nicotinamide (NA) is one form of niacin. A deficiency of this vitamin leads to loss of copper from the body, known as pellagra disease. Victims of pellagra show unusually high serum and urinary copper levels (Krishnamachari, 1974 ▸). The nicotinic acid derivative *N*,*N*-di­ethyl­nicotinamide (DENA) is an important respiratory stimulant (Bigoli *et al.*, 1972 ▸). The structures of some complexes obtained from the reactions of transition metal(II) ions with NA and DENA as ligands, *e.g*. [Ni(NA)_2_(C_7_H_4_ClO_2_)_2_(H_2_O)_2_] (Hökelek *et al.*, 2009*a* ▸) and [Ni(C_7_H_4_ClO_2_)_2_(C_10_H_14_N_2_O)_2_(H_2_O)_2_] (Hökelek *et al.*, 2009*b* ▸), have been the subject of much inter­est in our laboratory.
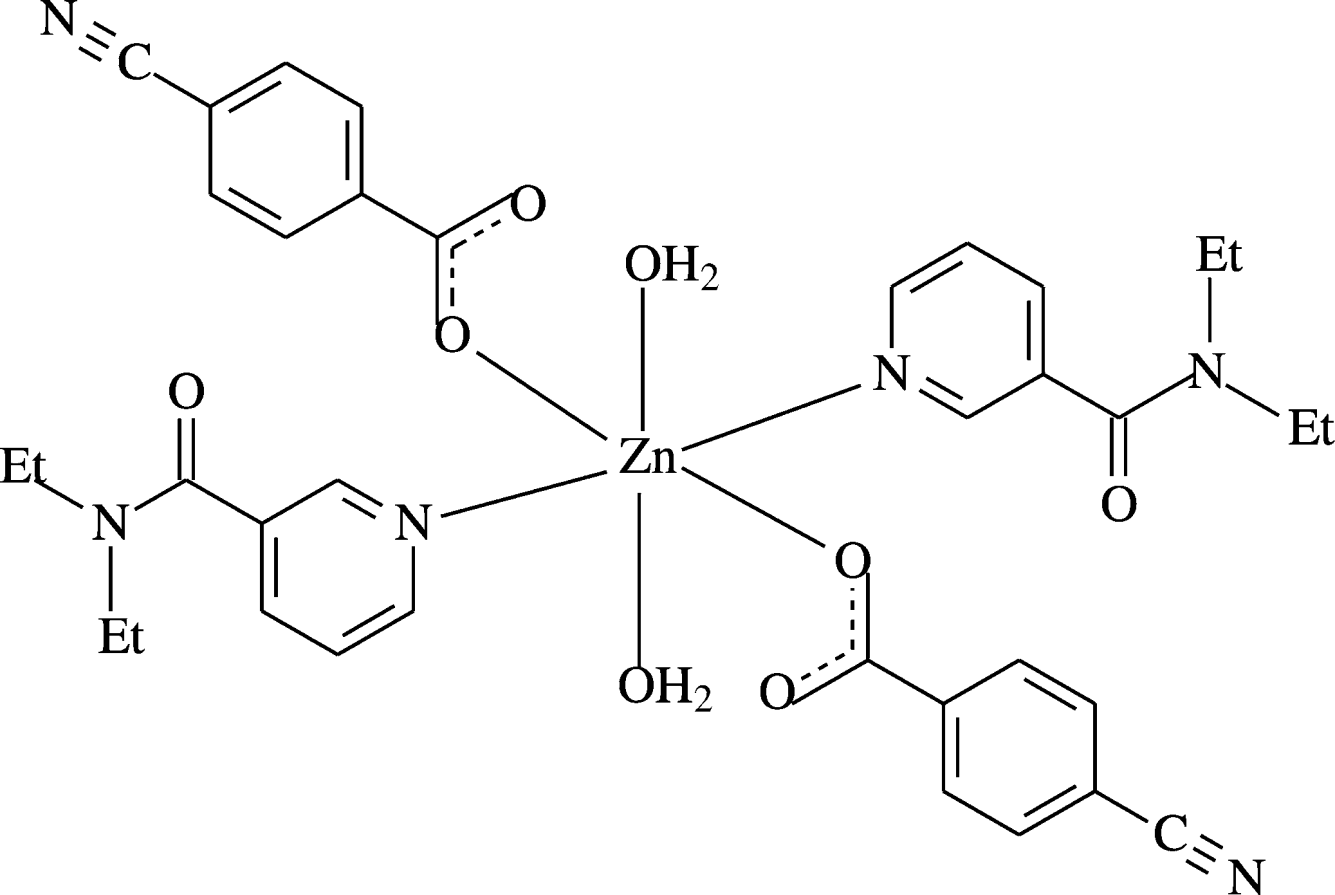


The structure-function–coordination relationships of the aryl­carboxyl­ate ion in Zn^II^ complexes of benzoic acid deriv­atives may change depending on the nature and position of the substituted groups on the benzene ring, the nature of the additional ligand mol­ecule or solvent, and the pH and temperature of synthesis (Shnulin *et al.*, 1981 ▸; Nadzhafov *et al.*, 1981 ▸; Antsyshkina *et al.*, 1980 ▸; Adiwidjaja *et al.*, 1978 ▸). When pyridine and its derivatives are used instead of water mol­ecules, the structure is completely different (Catterick *et al.*, 1974 ▸). In this context, we synthesized a Zn^II^-containing compound with 4-cyano­benzoate (CB) and DENA ligands, namely *trans*-di­aqua­bis­(4-cyano­benzoato-κ*O*)bis­(*N*,*N*-di­ethyl­nicotinamide-κ*N*)zinc(II), [Zn(DENA)_2_(CB)_2_(H_2_O)_2_], and report herein its crystal structure.

## Structural commentary   

**Figure 1:**
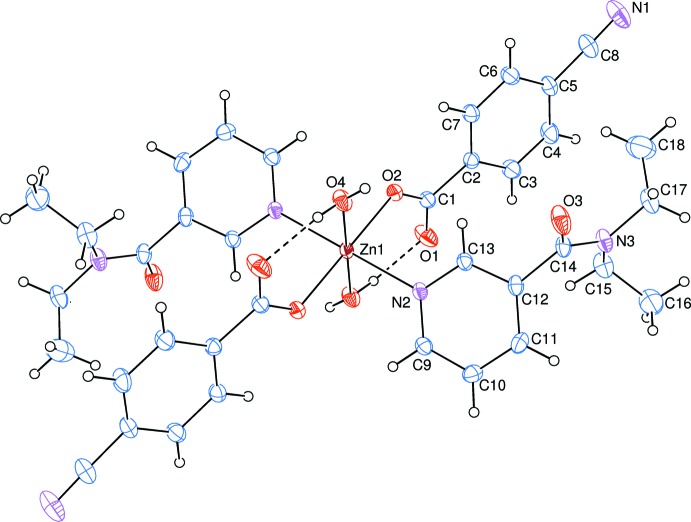
The mol­ecular structure of the title complex, showing the atom-numbering scheme for the asymmetric unit. Unlabelled atoms are generated by the symmetry operation −*x*, −*y*, −*z*. Displacement ellipsoids are drawn at the 40% probability level. Intra­molecular O—H_w_⋯O_c_ (w = water and c = non-coordinating carboxyl­ate O atom) hydrogen bonds, enclosing *S*(6) hydrogen-bonding motifs, are shown as dashed lines.

The asymmetric unit of the crystal structure of the title complex contains one Zn^II^ atom located on an inversion centre, one 4-cyano­benzoate (CB) ligand, one *N*,*N*-di­ethyl­nicotinamide (DENA) ligand and one water mol­ecule, all ligands coordinating to the Zn^II^ atom in a monodentate manner (Fig. 1 ▸).

The two carboxyl­ate O atoms (O2 and O2^i^) of the two symmetry-related monodentate CB anions and the two symmetry-related water O atoms (O4 and O4^i^) around the Zn1 atom form a slightly distorted square-planar arrangement, while the slightly distorted octa­hedral coordination sphere is completed by the two pyridine N atoms (N2 and N2^i^) of the two symmetry-related monodentate DENA ligands in the axial positions [symmetry code: (i) −*x*, −*y*, −*z*] (Fig. 1 ▸).

In the carboxyl­ate groups, the C—O bonds for coordinating O atoms are 0.0148 (19) Å longer than those of the non-coordinating ones [C1—O1 = 1.2436 (19) Å and C1—O2 = 1.2584 (18) Å], indicating delocalized bonding arrangements rather than localized single and double bonds. The Zn—O bond lengths are 2.1503 (11) Å (for water O atoms) and 2.0842 (10) Å (for benzoate O atoms) and the Zn—N bond length is 2.1501 (11) Å, the Zn—O bond lengths for water oxygen atoms are *ca* 0.07 Å longer than those involving the benzoate oxygen atoms. The Zn1 atom lies 0.7093 (1) Å below the planar (O1/O2/C1) carboxyl­ate group. The O—Zn—O and O–Zn—N bond angles range from 87.64 (5) to 92.36 (5)°.

The dihedral angle between the planar carboxyl­ate group (O1/O2/C1) and the adjacent benzene ring (C2–C7) is 9.50 (14)°, while the benzene and pyridine (N2/C9–C14) rings are oriented at a dihedral angle of 56.99 (5)°.

## Supra­molecular features   

**Figure 2:**
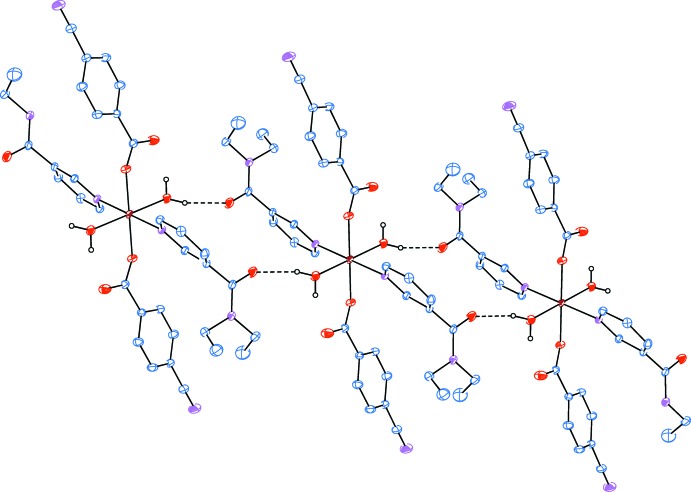
Part of the supra­molecular chain of the title compound. Inter­molecular O—H_w_⋯O_DENA_ (O_DENA_ = carbonyl O atom of *N*,*N*-di­ethyl­nicotin­amide) hydrogen bonds, enclosing 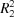
(16) ring motifs, are shown as dashed lines. The non-bonding H atoms have been omitted for clarity.

**Figure 3:**
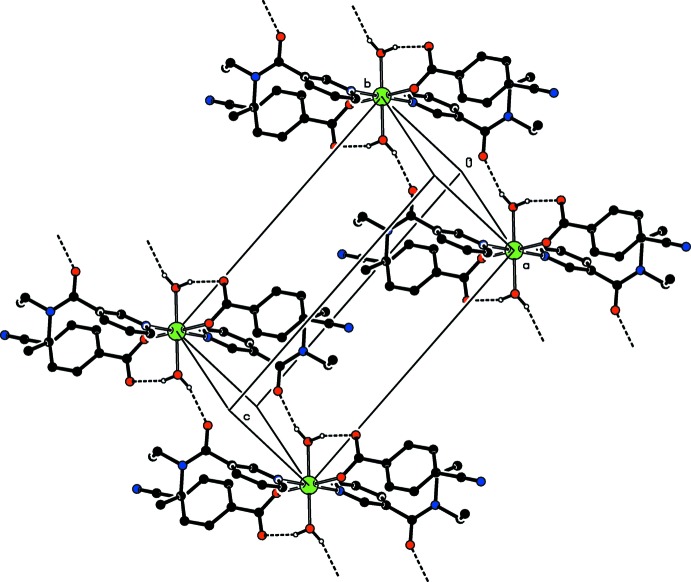
Part of the crystal structure. Intra- and inter­molecular (O—H_w_⋯O_c_ and O—H_w_⋯O_DENA_, respectively) hydrogen bonds are shown as dashed lines (see Table 1 ▸). The non-bonding H atoms have been omitted for clarity.

Intra­molecular O—H_w_⋯O_c_ (w = water, c = non-coordinating carboxyl­ate O atom) hydrogen bonds (Table 1 ▸) link two of the water ligands to the CB anions, enclosing *S*(6) hydrogen-bonding motifs (Fig. 1 ▸). The other water H atom is involved in inter­molecular O—H_w_⋯O_DENA_ (O_DENA_ = carbonyl O atom of *N*,*N*-di­ethyl­nicotinamide) hydrogen bonds (Table 1 ▸), enclosing 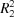
(16) ring motifs and leading to the formation of infinite chains (Fig. 2 ▸) propagating along the [110] direction (Fig. 3 ▸).

## Synthesis and crystallization   

The title compound was prepared by the reaction of ZnSO_4_·7H_2_O (1.44 g, 5 mmol) in H_2_O (50 ml) and di­ethyl­nicotinamide (1.78 g, 10 mmol) in H_2_O (10 ml) with sodium 4-cyano­benzoate (1.69 g, 10 mmol) in H_2_O (100 ml). The mixture was filtered and set aside to crystallize at ambient temperature for several days, giving translucent intense colourless single crystals.

## Refinement   

The experimental details including the crystal data, data collection and refinement are summarized in Table 2 ▸. Atoms H41 and H42 (for H_2_O) were located in a difference Fourier map and were refined freely. The C-bound H atoms were positioned geometrically with C—H = 0.93, 0.97 and 0.96 Å, for aromatic, methyl­ene and methyl H atoms, respectively, and constrained to ride on their parent atoms, with *U*_iso_(H) = *k* × *U*_eq_(C), where *k* = 1.5 for methyl H atoms and *k* = 1.2 for aromatic and methyl­ene H atoms.

## Supplementary Material

Crystal structure: contains datablock(s) I, global. DOI: 10.1107/S2056989016013815/xu5890sup1.cif

Structure factors: contains datablock(s) I. DOI: 10.1107/S2056989016013815/xu5890Isup2.hkl

CCDC reference: 1501337

Additional supporting information: crystallographic information; 3D view; checkCIF report

## Figures and Tables

**Table 1:** Hydrogen-bond geometry (Å, °)

*D*—H⋯*A*	*D*—H	H⋯*A*	*D*⋯*A*	*D*—H⋯*A*
O4—H41⋯O1^i^	0.83 (2)	1.84 (2)	2.6419 (19)	161 (2)
O4—H42⋯O3^ii^	0.82 (2)	2.03 (2)	2.827 (2)	163 (2)

Symmetry codes: (i) 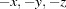; (ii) 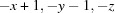.

**Table 2:** Experimental details

Crystal data	
Chemical formula	[Zn(C_8_H_4_NO_2_)_2_(C_10_H_14_N_2_O)_2_(H_2_O)_2_]
*M*_r_	750.13
Crystal system, space group	Triclinic, *P* 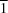
Temperature (K)	296
*a*, *b*, *c* (Å)	7.4916 (3), 8.5915 (3), 15.0343 (6)
α, β, γ (°)	86.363 (3), 75.894 (2), 74.390 (2)
*V* (Å^3^)	903.87 (6)
*Z*	1
Radiation type	Mo *K*α
μ (mm^−1^)	0.74
Crystal size (mm)	0.45 × 0.30 × 0.24

Data collection	
Diffractometer	Bruker SMART BREEZE CCD
Absorption correction	Multi-scan (*SADABS*; Bruker, 2012 ▸)
*T*_min_, *T*_max_	0.74, 0.84
No. of measured, independent and observed [*I* > 2σ(*I*)] reflections	19149, 4366, 4029
*R*_int_	0.020
(sin θ/λ)_max_ (Å^−1^)	0.666

Refinement	
*R*[*F* ^2^ > 2σ(*F* ^2^)], *wR*(*F* ^2^), *S*	0.032, 0.084, 1.06
No. of reflections	4366
No. of parameters	242
H-atom treatment	H atoms treated by a mixture of independent and constrained refinement
Δρ_max_, Δρ_min_ (e Å^−3^)	0.56, −0.23

Computer programs: *APEX2* and *SAINT* (Bruker, 2012 ▸), *SHELXS97* and *SHELXL97* (Sheldrick, 2008 ▸), *ORTEP-3 for Windows* and *WinGX* (Farrugia, 2012 ▸) and *PLATON* (Spek, 2009 ▸).

## supplementary crystallographic information

### Crystal data

**Table:**

[Zn(C_8_H_4_NO_2_)_2_(C_10_H_14_N_2_O)_2_(H_2_O)_2_]	*Z* = 1
*M**_r_* = 750.13	*F*(000) = 392
Triclinic, *P*1	*D*_x_ = 1.378 Mg m^−^^3^
*a* = 7.4916 (3) Å	Mo *K*α radiation, λ = 0.71073 Å
*b* = 8.5915 (3) Å	Cell parameters from 9994 reflections
*c* = 15.0343 (6) Å	θ = 2.8–28.2°
α = 86.363 (3)°	µ = 0.74 mm^−^^1^
β = 75.894 (2)°	*T* = 296 K
γ = 74.390 (2)°	Prism, translucent intense colourless
*V* = 903.87 (6) Å^3^	0.45 × 0.30 × 0.24 mm

### Data collection

**Table:**

Bruker SMART BREEZE CCD diffractometer	4366 independent reflections
Radiation source: fine-focus sealed tube	4029 reflections with *I* > 2σ(*I*)
Graphite monochromator	*R*_int_ = 0.020
φ and ω scans	θ_max_ = 28.3°, θ_min_ = 1.4°
Absorption correction: multi-scan (SADABS; Bruker, 2012)	*h* = −9→9
*T*_min_ = 0.74, *T*_max_ = 0.84	*k* = −11→11
19149 measured reflections	*l* = −19→19

### Refinement

**Table:**

Refinement on *F*^2^	Primary atom site location: structure-invariant direct methods
Least-squares matrix: full	Secondary atom site location: difference Fourier map
*R*[*F*^2^ > 2σ(*F*^2^)] = 0.032	Hydrogen site location: inferred from neighbouring sites
*wR*(*F*^2^) = 0.084	H atoms treated by a mixture of independent and constrained refinement
*S* = 1.06	*w* = 1/[σ^2^(*F*_o_^2^) + (0.0458*P*)^2^ + 0.2769*P*] where *P* = (*F*_o_^2^ + 2*F*_c_^2^)/3
4366 reflections	(Δ/σ)_max_ < 0.001
242 parameters	Δρ_max_ = 0.56 e Å^−^^3^
0 restraints	Δρ_min_ = −0.23 e Å^−^^3^

### Special details

**Table:**

Geometry. All esds (except the esd in the dihedral angle between two l.s. planes) are estimated using the full covariance matrix. The cell esds are taken into account individually in the estimation of esds in distances, angles and torsion angles; correlations between esds in cell parameters are only used when they are defined by crystal symmetry. An approximate (isotropic) treatment of cell esds is used for estimating esds involving l.s. planes.
Refinement. Refinement of F^2^ against ALL reflections. The weighted R-factor wR and goodness of fit S are based on F^2^, conventional R-factors R are based on F, with F set to zero for negative F^2^. The threshold expression of F^2^ > 2sigma(F^2^) is used only for calculating R-factors(gt) etc. and is not relevant to the choice of reflections for refinement. R-factors based on F^2^ are statistically about twice as large as those based on F, and R- factors based on ALL data will be even larger.

### Fractional atomic coordinates and isotropic or equivalent isotropic displacement parameters (Å^2^)

**Table:**

	*x*	*y*	*z*	*U*_iso_*/*U*_eq_	
Zn1	0.0000	0.0000	0.0000	0.02730 (8)	
O1	0.10666 (18)	0.0679 (2)	−0.22778 (9)	0.0585 (4)	
O2	0.23197 (15)	0.01976 (13)	−0.10544 (7)	0.0340 (2)	
O3	0.51295 (19)	−0.62718 (18)	−0.11834 (9)	0.0571 (4)	
O4	0.19075 (17)	−0.10842 (16)	0.08623 (9)	0.0414 (3)	
H41	0.115 (3)	−0.110 (3)	0.1366 (17)	0.056 (7)*	
H42	0.277 (3)	−0.191 (3)	0.0848 (16)	0.059 (7)*	
N1	1.1465 (3)	−0.2028 (3)	−0.46622 (14)	0.0755 (6)	
N2	0.02013 (16)	−0.22996 (14)	−0.05596 (8)	0.0288 (2)	
N3	0.4843 (2)	−0.58488 (17)	−0.26504 (9)	0.0402 (3)	
C1	0.2428 (2)	0.02304 (18)	−0.19037 (10)	0.0324 (3)	
C2	0.4408 (2)	−0.03327 (17)	−0.25173 (10)	0.0304 (3)	
C3	0.4653 (2)	−0.0552 (2)	−0.34513 (11)	0.0423 (4)	
H3	0.3593	−0.0388	−0.3698	0.051*	
C4	0.6461 (3)	−0.1012 (2)	−0.40159 (11)	0.0466 (4)	
H4	0.6621	−0.1157	−0.4641	0.056*	
C5	0.8044 (2)	−0.12572 (19)	−0.36423 (11)	0.0383 (3)	
C6	0.7815 (2)	−0.1071 (2)	−0.27117 (12)	0.0415 (4)	
H6	0.8876	−0.1252	−0.2463	0.050*	
C7	0.5995 (2)	−0.0614 (2)	−0.21525 (11)	0.0368 (3)	
H7	0.5836	−0.0493	−0.1525	0.044*	
C8	0.9951 (3)	−0.1689 (2)	−0.42235 (13)	0.0507 (4)	
C9	−0.1333 (2)	−0.28196 (18)	−0.05414 (10)	0.0323 (3)	
H9	−0.2528	−0.2183	−0.0253	0.039*	
C10	−0.1218 (2)	−0.4269 (2)	−0.09346 (12)	0.0379 (3)	
H10	−0.2314	−0.4600	−0.0909	0.045*	
C11	0.0555 (2)	−0.52178 (18)	−0.13656 (11)	0.0386 (3)	
H11	0.0669	−0.6187	−0.1644	0.046*	
C12	0.2158 (2)	−0.47000 (17)	−0.13758 (10)	0.0317 (3)	
C13	0.1916 (2)	−0.32445 (17)	−0.09573 (10)	0.0302 (3)	
H13	0.2994	−0.2907	−0.0952	0.036*	
C14	0.4178 (2)	−0.56899 (17)	−0.17441 (11)	0.0354 (3)	
C15	0.3800 (3)	−0.5002 (2)	−0.33283 (12)	0.0488 (4)	
H15A	0.4514	−0.4299	−0.3693	0.059*	
H15B	0.2577	−0.4329	−0.3008	0.059*	
C16	0.3474 (3)	−0.6144 (3)	−0.39581 (16)	0.0663 (6)	
H16A	0.2720	−0.5537	−0.4359	0.099*	
H16B	0.2815	−0.6874	−0.3600	0.099*	
H16C	0.4680	−0.6748	−0.4317	0.099*	
C17	0.6830 (3)	−0.6752 (2)	−0.30132 (13)	0.0481 (4)	
H17A	0.6893	−0.7410	−0.3527	0.058*	
H17B	0.7267	−0.7473	−0.2542	0.058*	
C18	0.8137 (4)	−0.5660 (4)	−0.3324 (2)	0.0831 (8)	
H18A	0.9426	−0.6304	−0.3527	0.125*	
H18B	0.8053	−0.4984	−0.2823	0.125*	
H18C	0.7766	−0.4997	−0.3821	0.125*	

### Atomic displacement parameters (Å^2^)

**Table:**

	*U*^11^	*U*^22^	*U*^33^	*U*^12^	*U*^13^	*U*^23^
Zn1	0.02409 (12)	0.02632 (12)	0.02797 (13)	−0.00158 (8)	−0.00370 (8)	−0.00585 (8)
O1	0.0317 (6)	0.0954 (11)	0.0390 (7)	−0.0015 (6)	−0.0080 (5)	0.0024 (7)
O2	0.0289 (5)	0.0415 (6)	0.0297 (5)	−0.0098 (4)	−0.0014 (4)	−0.0061 (4)
O3	0.0501 (7)	0.0621 (8)	0.0419 (7)	0.0212 (6)	−0.0165 (6)	−0.0093 (6)
O4	0.0310 (6)	0.0488 (7)	0.0375 (6)	0.0048 (5)	−0.0113 (5)	−0.0020 (5)
N1	0.0492 (10)	0.0958 (16)	0.0549 (11)	0.0045 (10)	0.0111 (9)	−0.0003 (10)
N2	0.0268 (6)	0.0263 (5)	0.0297 (6)	−0.0019 (4)	−0.0049 (5)	−0.0032 (4)
N3	0.0392 (7)	0.0349 (7)	0.0353 (7)	0.0061 (5)	−0.0050 (6)	−0.0021 (5)
C1	0.0295 (7)	0.0314 (7)	0.0343 (7)	−0.0074 (5)	−0.0040 (6)	−0.0017 (6)
C2	0.0314 (7)	0.0303 (7)	0.0284 (7)	−0.0094 (5)	−0.0033 (6)	−0.0001 (5)
C3	0.0372 (8)	0.0564 (10)	0.0321 (8)	−0.0091 (7)	−0.0094 (6)	−0.0015 (7)
C4	0.0471 (10)	0.0597 (11)	0.0266 (7)	−0.0070 (8)	−0.0034 (7)	−0.0035 (7)
C5	0.0343 (8)	0.0374 (8)	0.0349 (8)	−0.0048 (6)	0.0025 (6)	−0.0014 (6)
C6	0.0318 (8)	0.0527 (10)	0.0379 (8)	−0.0086 (7)	−0.0059 (6)	−0.0035 (7)
C7	0.0334 (8)	0.0469 (9)	0.0287 (7)	−0.0099 (6)	−0.0040 (6)	−0.0046 (6)
C8	0.0421 (10)	0.0553 (11)	0.0406 (9)	−0.0013 (8)	0.0036 (8)	0.0016 (8)
C9	0.0281 (7)	0.0340 (7)	0.0323 (7)	−0.0042 (5)	−0.0067 (6)	−0.0002 (6)
C10	0.0364 (8)	0.0381 (8)	0.0436 (9)	−0.0124 (6)	−0.0148 (7)	0.0002 (7)
C11	0.0466 (9)	0.0285 (7)	0.0422 (8)	−0.0065 (6)	−0.0158 (7)	−0.0061 (6)
C12	0.0349 (7)	0.0258 (6)	0.0296 (7)	0.0007 (5)	−0.0077 (6)	−0.0015 (5)
C13	0.0276 (7)	0.0275 (6)	0.0323 (7)	−0.0032 (5)	−0.0052 (5)	−0.0024 (5)
C14	0.0373 (8)	0.0257 (6)	0.0367 (8)	0.0032 (6)	−0.0080 (6)	−0.0054 (6)
C15	0.0511 (10)	0.0477 (10)	0.0367 (9)	0.0030 (8)	−0.0089 (8)	0.0050 (7)
C16	0.0596 (13)	0.0810 (16)	0.0555 (12)	−0.0073 (11)	−0.0190 (10)	−0.0058 (11)
C17	0.0395 (9)	0.0476 (9)	0.0434 (9)	0.0054 (7)	−0.0015 (7)	−0.0057 (8)
C18	0.0576 (14)	0.099 (2)	0.093 (2)	−0.0287 (14)	−0.0102 (13)	0.0008 (16)

### Geometric parameters (Å, º)

**Table:**

Zn1—O2	2.0842 (10)	C6—H6	0.9300
Zn1—O2^i^	2.0842 (10)	C7—C6	1.384 (2)
Zn1—O4	2.1503 (11)	C7—H7	0.9300
Zn1—O4^i^	2.1503 (11)	C8—N1	1.135 (3)
Zn1—N2	2.1501 (11)	C9—C10	1.385 (2)
Zn1—N2^i^	2.1501 (11)	C9—H9	0.9300
O1—C1	1.2436 (19)	C10—H10	0.9300
O2—C1	1.2584 (18)	C11—C10	1.383 (2)
O3—C14	1.231 (2)	C11—H11	0.9300
O4—H41	0.83 (2)	C12—C11	1.385 (2)
O4—H42	0.82 (2)	C12—C14	1.509 (2)
N2—C9	1.3348 (19)	C13—C12	1.384 (2)
N2—C13	1.3389 (17)	C13—H13	0.9300
N3—C14	1.334 (2)	C15—C16	1.510 (3)
N3—C15	1.472 (2)	C15—H15A	0.9700
N3—C17	1.467 (2)	C15—H15B	0.9700
C1—C2	1.513 (2)	C16—H16A	0.9600
C2—C3	1.389 (2)	C16—H16B	0.9600
C2—C7	1.386 (2)	C16—H16C	0.9600
C3—C4	1.380 (2)	C17—C18	1.508 (3)
C3—H3	0.9300	C17—H17A	0.9700
C4—H4	0.9300	C17—H17B	0.9700
C5—C4	1.393 (2)	C18—H18A	0.9600
C5—C6	1.382 (2)	C18—H18B	0.9600
C5—C8	1.446 (2)	C18—H18C	0.9600
			
O2—Zn1—O2^i^	180.00 (7)	C6—C7—H7	119.7
O2—Zn1—O4	89.94 (5)	N1—C8—C5	178.4 (2)
O2^i^—Zn1—O4	90.06 (5)	N2—C9—C10	122.61 (14)
O2—Zn1—O4^i^	90.06 (5)	N2—C9—H9	118.7
O2^i^—Zn1—O4^i^	89.94 (5)	C10—C9—H9	118.7
O2—Zn1—N2	88.48 (4)	C9—C10—H10	120.6
O2^i^—Zn1—N2	91.52 (4)	C11—C10—C9	118.88 (14)
O2—Zn1—N2^i^	91.52 (4)	C11—C10—H10	120.6
O2^i^—Zn1—N2^i^	88.48 (4)	C10—C11—C12	118.92 (14)
O4^i^—Zn1—O4	180.00 (10)	C10—C11—H11	120.5
N2—Zn1—O4	92.36 (5)	C12—C11—H11	120.5
N2^i^—Zn1—O4	87.64 (5)	C11—C12—C14	124.06 (13)
N2—Zn1—O4^i^	87.64 (5)	C13—C12—C11	118.44 (13)
N2^i^—Zn1—O4^i^	92.36 (5)	C13—C12—C14	117.28 (13)
N2^i^—Zn1—N2	180.00 (6)	N2—C13—C12	122.99 (13)
C1—O2—Zn1	127.55 (9)	N2—C13—H13	118.5
Zn1—O4—H41	101.6 (16)	C12—C13—H13	118.5
Zn1—O4—H42	135.9 (16)	O3—C14—N3	123.94 (14)
H41—O4—H42	106 (2)	O3—C14—C12	117.46 (14)
C9—N2—Zn1	122.37 (9)	N3—C14—C12	118.58 (13)
C9—N2—C13	118.12 (12)	N3—C15—C16	112.80 (17)
C13—N2—Zn1	119.50 (9)	N3—C15—H15A	109.0
C14—N3—C15	124.36 (14)	N3—C15—H15B	109.0
C14—N3—C17	118.81 (14)	C16—C15—H15A	109.0
C17—N3—C15	116.34 (14)	C16—C15—H15B	109.0
O1—C1—O2	126.05 (14)	H15A—C15—H15B	107.8
O1—C1—C2	117.65 (14)	C15—C16—H16A	109.5
O2—C1—C2	116.30 (13)	C15—C16—H16B	109.5
C3—C2—C1	120.47 (14)	C15—C16—H16C	109.5
C7—C2—C3	119.42 (14)	H16A—C16—H16B	109.5
C7—C2—C1	120.10 (13)	H16A—C16—H16C	109.5
C2—C3—H3	119.8	H16B—C16—H16C	109.5
C4—C3—C2	120.43 (15)	N3—C17—C18	112.50 (18)
C4—C3—H3	119.8	N3—C17—H17A	109.1
C3—C4—C5	119.49 (15)	N3—C17—H17B	109.1
C3—C4—H4	120.3	C18—C17—H17A	109.1
C5—C4—H4	120.3	C18—C17—H17B	109.1
C4—C5—C8	120.56 (16)	H17A—C17—H17B	107.8
C6—C5—C4	120.54 (15)	C17—C18—H18A	109.5
C6—C5—C8	118.90 (16)	C17—C18—H18B	109.5
C5—C6—C7	119.41 (15)	C17—C18—H18C	109.5
C5—C6—H6	120.3	H18A—C18—H18B	109.5
C7—C6—H6	120.3	H18A—C18—H18C	109.5
C2—C7—H7	119.7	H18B—C18—H18C	109.5
C6—C7—C2	120.68 (15)		
			
O4—Zn1—O2—C1	153.35 (13)	C15—N3—C17—C18	73.3 (2)
O4^i^—Zn1—O2—C1	−26.65 (13)	O1—C1—C2—C3	−8.8 (2)
N2—Zn1—O2—C1	60.99 (12)	O1—C1—C2—C7	170.29 (16)
N2^i^—Zn1—O2—C1	−119.01 (12)	O2—C1—C2—C3	171.11 (15)
O2—Zn1—N2—C9	−144.71 (12)	O2—C1—C2—C7	−9.8 (2)
O2^i^—Zn1—N2—C9	35.29 (12)	C1—C2—C3—C4	177.77 (16)
O2—Zn1—N2—C13	34.08 (11)	C7—C2—C3—C4	−1.4 (3)
O2^i^—Zn1—N2—C13	−145.92 (11)	C1—C2—C7—C6	−177.60 (15)
O4—Zn1—N2—C9	125.41 (12)	C3—C2—C7—C6	1.5 (2)
O4^i^—Zn1—N2—C9	−54.59 (12)	C2—C3—C4—C5	0.0 (3)
O4—Zn1—N2—C13	−55.80 (11)	C6—C5—C4—C3	1.2 (3)
O4^i^—Zn1—N2—C13	124.20 (11)	C8—C5—C4—C3	−178.01 (18)
Zn1—O2—C1—O1	25.4 (2)	C4—C5—C6—C7	−1.0 (3)
Zn1—O2—C1—C2	−154.52 (10)	C8—C5—C6—C7	178.20 (17)
Zn1—N2—C9—C10	177.24 (12)	C2—C7—C6—C5	−0.4 (3)
C13—N2—C9—C10	−1.6 (2)	N2—C9—C10—C11	−0.1 (2)
Zn1—N2—C13—C12	−176.49 (11)	C12—C11—C10—C9	1.1 (2)
C9—N2—C13—C12	2.3 (2)	C13—C12—C11—C10	−0.4 (2)
C15—N3—C14—O3	−172.38 (18)	C14—C12—C11—C10	174.11 (15)
C15—N3—C14—C12	5.8 (2)	C11—C12—C14—O3	−107.28 (19)
C17—N3—C14—O3	−0.7 (3)	C11—C12—C14—N3	74.5 (2)
C17—N3—C14—C12	177.46 (14)	C13—C12—C14—O3	67.3 (2)
C14—N3—C15—C16	−121.41 (19)	C13—C12—C14—N3	−111.00 (17)
C17—N3—C15—C16	66.7 (2)	N2—C13—C12—C11	−1.4 (2)
C14—N3—C17—C18	−99.1 (2)	N2—C13—C12—C14	−176.26 (13)

Symmetry code: (i) −*x*, −*y*, −*z*.

### Hydrogen-bond geometry (Å, º)

**Table:**

*D*—H···*A*	*D*—H	H···*A*	*D*···*A*	*D*—H···*A*
O4—H41···O1^i^	0.83 (2)	1.84 (2)	2.6419 (19)	161 (2)
O4—H42···O3^ii^	0.82 (2)	2.03 (2)	2.827 (2)	163 (2)

Symmetry codes: (i) −*x*, −*y*, −*z*; (ii) −*x*+1, −*y*−1, −*z*.
